# Supplementary material for: BTW—Bioinformatics Through Windows: an easy-to-install package to analyze marker gene data
Source: PeerJ. 2018 Jul 30;6:e5299. doi: 10.7717/peerj.5299 (PMC6074753; doi:10.7717/peerj.5299)
Supplement: Supplemental Information 2 [file peerj-06-5299-s002.pdf]

BTW – Bioinformatics Through Windows: an easy-to-install package to analyze marker gene data

Documentation

Installing BTW

BTW package installation instructions can be found at http://www.brmicrobiome.org/tutorialbtw (steps 6 and 7).

Installing the BTW package

Open the installed Ubuntu on Windows.

- In the terminal, type:

```
wget https://raw.githubusercontent.com/vpylro/BTW/master/win_bmp.sh
```

- Then type:

```
sudo bash win_bmp.sh
```

Your UNIX account password will be requested. Type your password and then ENTER. The BTW package will be installed automatically. This may take several minutes, depending on your computer and internet connection speeds.

After finish installing, close the Ubuntu and open it again. The BMP recommended pipeline for 16S rRNA data analyses is available at http://www.brmicrobiome.org/win16s

16S profiling analysis pipeline (Windows)

BMP advisory board recommend the use of this pipeline as a standard for 16S rRNA data analysis.

We are now working in order to improve this workflow besides making it easier for end-users.

If you have any questions or suggestions, please contact ***Victor Pylro: victor.pylro@brmicrobiome.org, Daniel Morais: or Luiz Roesch: luiz.roesch@brmicrobiome.org***

Please, cite our efforts when using this pipeline: **Data analysis for 16S microbial profiling from different benchtop sequencing platforms.** ***J Microbiol Methods.*** **2014. doi: 10.1016/j.mimet.2014.08.018.**

Please, cite our efforts when using the BTW package: **BTW – Bioinformatics Through Windows: an easy-to-install package to analyze marker gene data.** ***(Submitted)***

Also, remember to cite all other programs applied here.

VSEARCH (version 2.8.0) https://github.com/torognes/vsearch

Rognes T, Flouri T, Nichols B, Quince C, Mahé F. (2016) VSEARCH: a versatile open source tool for metagenomics. PeerJ 4:e2584. doi: 10.7717/peerj.2584

QIIME (version 1.9.1) http://www.qiime.org

Caporaso J G, et al. (2010) QIIME allows analysis of high-throughput community sequencing data. Nature Methods, doi:10.1038/nmeth.f.303

FastX Toolkit (version 0.0.14) http://hannonlab.cshl.edu/fastx_toolkit/

fastq-join (version 1.3.1) https://github.com/brwnj/fastq-join

Aronesty E. (2013). TOBioiJ: Comparison of Sequencing Utility Programs. DOI:10.2174/1875036201307010001

FLASH (version 1.2.11) https://ccb.jhu.edu/software/FLASH/

Magoc T, Salzberg S. (2011) FLASH: Fast length adjustment of short reads to improve genome assemblies. Bioinformatics 27:21, 2957-2963.

ClustalW (version 2.1)

RDP Classifier (version 2.2) http://qiime.org/install/alternative.html#rdp-install

Here, we provide the recommended pipeline for 16S profiling analysis using the Windows Subsystem for Linux (WSL)

What you need: only the BTW package!! Click here

Installing the Windows Subsystem for Linux (WSL): Click here

This example assumes reads in **FASTQ format**.

This page gives a complete pipeline to analyze 16S rRNA gene data. Of course, you should edit as needed for your reads and file locations (represented here as $PWD/).

From Illumina paired-end reads: this pipelines assumes that you have an input folder of paired-up files (by filename, with the default _R1_ and _R2_ containing the forward and reverse reads filenames, respectively):

1 - Take forward and reverse Illumina reads (R1.fastq and R2.fastq files) and join them using the method *fastq-join* **<<<USING QIIME 1.9>>>**

```
multiple_join_paired_ends.py -i input_files -o merged/
```

1.1 - Alternatively, FLASH can be applied to perform the same task (this must be done for each sample, separately)

```
flash -m 20 -M 250 -x 0.25 -p 33 R1.fastq R2.fastq -o merged/
```

2 - Quality filtering, length truncate, and convert to FASTA each joined sample **<<<USING VSEARCH>>>**

```
vsearch --fastx_filter   $PWD/fastqjoin.join.fastq   --fastq_maxee   1.0 --
fastq_trunclen 220 --fastaout samplex.fa
```

Obs: the `--fastq_trunclen` parameter will depend on the length of you joined reads. You can use **FASTQC** to taking this decision.

3 - Change sequence header to make file compatible with further steps **<<<USING BMP PERL SCRIPT>>>**. This script will generate your converted FASTA file. *Sample´s name should not contain any special characters, symbols or spaces. We strongly recommend keeping samples´s name as simple as possible.*

```
bmp_demultiplexed.pl -i samplex.fa -o samplename -b samplename
```

4 - Make a single file containing all your samples

```
cat sample1 sample2 sample3 sample4 ... > reads.fa
```

5 - Dereplication **<<<USING VSEARCH>>>**

```
vsearch --derep_fulllength $PWD/reads.fa --output derep.fa --sizeout
```

6 - Abundance sort and discard singletons **<<<USING VSEARCH>>>**

```
vsearch --sortbysize $PWD/derep.fa --output sorted.fa --minsize 2
```

7 - OTU clustering using UPARSE method **<<<USING VSEARCH>>>**

```
vsearch --cluster_size $PWD/sorted.fa --consout otus1.fa --id 0.97
```

8 - Fasta Formatter **<<<FASTX TOOLKIT SCRIPT>>>**

```
fasta_formatter -i otus1.fa -o formated_otus1.fa
```

9 - Renamer **<<<BMP SCRIPT>>>**

```
bmp-otuName.pl -i formated_otus1.fa -o otus.fa
```

10 - Map reads back to OTU database **<<<VSEARCH>>>**

```
vsearch --usearch_global $PWD/reads.fa --db otus.fa --strand plus --id 0.97
--uc map.txt
```

11 - Assign taxonomy to OTUS using the RDP Classifier **on QIIME** (use the file “otus.fa” as input file)

```
assign_taxonomy.py -i $PWD/otus.fa -m rdp -o taxonomy
```

12 - Align sequences **on QIIME**, using the ClustalW method (use the file “otus.fa” as input file)

```
align_seqs.py -i $PWD/otus.fa -m clustalw -o rep_set_align
```

13 - Filter alignments **on QIIME**

```
filter_alignment.py -i $PWD/otus_aligned.fasta -o filtered_alignment
```

14 - Make the reference tree **on QIIME**

```
make_phylogeny.py -i $PWD/otus_aligned_pfiltered.fasta -o rep_set.tre
```

15 - Convert UC to otu-table.txt **<<< BMP SCRIPT>>>**

```
bmp-map2qiime.py map.uc > otu_table.txt
```

16 - Convert otu_table.txt to otu-table.biom **<<< QIIME SCRIPT>>>**

```
make_otu_table.py -i otu_table.txt -t otus_tax_assignments.txt -o
otu_table.biom
```

17 - Check OTU Table on QIIME.

```
biom summarize-table -i $PWD/otu_table.biom -o results_biom_table
```

18 - Run diversity analyses **on QIIME** (or any other analysis of your choice). The parameter “-e” is the sequencing depth to use for even sub-sampling and maximum rarefaction depth. You should review the output of the ‘biom summarize-table’ (step 18) command to decide on this value.

```
core_diversity_analyses.py -i $PWD/otu_table.biom -m $PWD/mapping_file.txt
-t $PWD/rep_set.tre -e xxxx -o $PWD/core_output
```

The generated .biom OTU table is also fully compatible with the MicrobiomeAnalyst, a user-friendly web-based platform for microbiome data analyses and visualizations, including taxonomy plots and estimates of α- and β-diversity (http://www.microbiomeanalyst.ca).

BTW Team

Daniel Kumazawa Morais (Institute of Microbiology of the CAS - Czech Republic)

Fausto Gonçalves dos Santos (René Rachou Research Center - Brazil)

Luiz Fernando Wurdig Roesch (Universidade Federal do Pampa - Brazil)

Marc Redmile-Gordon (Natural England - United Kingdom)

Petr Baldrian (Institute of Microbiology of the CAS - Czech Republic)

Fernando Dini Andreote (ESALQ/USP - Brazil)

Victor Satler Pylro (UFLA - Brazil)
